# Spike Timing-Dependent Plasticity in the Mouse Barrel Cortex Is Strongly Modulated by Sensory Learning and Depends on Activity of Matrix Metalloproteinase 9

**DOI:** 10.1007/s12035-016-0174-y

**Published:** 2016-10-15

**Authors:** Katarzyna Lebida, Jerzy W. Mozrzymas

**Affiliations:** 1 Laboratory of Neuroscience, Department of Biophysics, Wroclaw Medical University, Chalubinskiego 3a, 50-368 Wroclaw, Poland; 2 Department of Animal Molecular Physiology, Institute of Experimental Biology, Wroclaw University, Wroclaw, Poland

**Keywords:** Matrix metalloproteinases, Synaptic plasticity, Somatosensory cortex, Learning, Tonic inhibition

## Abstract

Experience and learning in adult primary somatosensory cortex are known to affect neuronal circuits by modifying both excitatory and inhibitory transmission. Synaptic plasticity phenomena provide a key substrate for cognitive processes, but precise description of the cellular and molecular correlates of learning is hampered by multiplicity of these mechanisms in various projections and in different types of neurons. Herein, we investigated the impact of associative learning on neuronal plasticity in distinct types of postsynaptic neurons by checking the impact of classical conditioning (pairing whisker stroking with tail shock) on the spike timing-dependent plasticity (t-LTP and t-LTD) in the layer IV to II/III vertical pathway of the mouse barrel cortex. Learning in this paradigm practically prevented t-LTP measured in pyramidal neurons but had no effect on t-LTD. Since classical conditioning is known to affect inhibition in the barrel cortex, we examined its effect on tonic GABAergic currents and found a strong downregulation of these currents in the layer II/III interneurons but not in pyramidal cells. Matrix metalloproteinases emerged as crucial players in synaptic plasticity and learning. We report that the blockade of MMP-9 (but not MMP-3) abolished t-LTP having no effect on t-LTD. Moreover, associative learning resulted in an upregulation of gelatinolytic activity within the “trained” barrel. We conclude that LTP induced by spike timing-dependent plasticity (STDP) paradigm is strongly correlated with associative learning and critically depends on the activity of MMP-9*.*

**Electronic supplementary material:**

The online version of this article (doi:10.1007/s12035-016-0174-y) contains supplementary material, which is available to authorized users.

## Introduction

It is well established that in adult primary somatosensory cortex neuronal circuits can be modified by experience and learning [1–4]. In particular, learning in classical conditioning paradigm, in which stimulation of vibrissae is paired with tail shock, is known to induce profound plastic changes in the mouse barrel cortex [5–9]. Interestingly, most prominent plastic changes were found in barrels representing whiskers stimulated during the conditioning and long-term changes concerned primarily the GABAergic system [10, 11]. In particular, modulation of this system is reflected by increased frequency of inhibitory postsynaptic currents in layer IV excitatory neurons [12] and by changes in tonic current intensity in a neuron-specific manner [13]. Moreover, in a recent study, Posluszny et al. [14] have shown that a downregulation of the inhibitory drive prevented both learning-induced enlargement of the conditioned vibrissae representation in murine barrel cortex and impaired the conditioned response in the behavioral experiments. On the other hand, behavioral learning does not result in a mere rescaling of inhibition. Indeed, Gdalyahu et al. [15] have found that associative learning decreased total network activity in primary somatosensory cortex but signaling from responsive neurons was increased, suggesting enhanced efficiency of sensory processing. Moreover, Rosselet et al. [16] have demonstrated that associative learning markedly affected the ascending layer IV excitatory projection to the pyramidal cells in the layer III.

In general, it is believed that phenomena of synaptic plasticity provide a key substrate for cognitive processes. However, understanding of cellular correlates of learning is hampered by a multiplicity of plasticity mechanisms occurring at various projections and at different types of neurons. To tackle this problem, spike timing-dependent plasticity (STDP) seems an appropriate tool. Indeed, in this paradigm, due to a precise time window between activity of pre- and postsynaptic neurons, well-defined long-lasting changes in synaptic weights can be induced and characterized for specific pathways and identified neurons [e.g., 17–21]. Interestingly, it was found that sensory deprivation affects STDP (both t-LTP and t-LTD) in the barrel cortex in vivo and in vitro [22–25]. However, whether or not classical conditioning affects the two basic forms of STDP—LTP and LTD—has not been systematically investigated. It seemed thus interesting to check the impact of associative learning on these two forms of plasticity.

In the past decade, an extensive body of evidence accumulated that matrix metalloproteinases play a crucial role in mechanisms of synaptic plasticity as well as in learning and memory in hippocampus, amygdala, and cortex [26–29]*.* Several lines of evidence indicate that especially MMP-9 and MMP-3 play a pivotal role in these processes [30–36]. More recently, it has been shown that in the cortex both MMPs are involved in learning and in the plasticity processes [36–39]. In particular, Kaliszewska et al. [38] have shown that MMP-9 knockout resulted in a decreased plasticity in layer IV and II/III in barrel cortex of adult mice. However, the effect of MMPs on STDP in the context of associative learning has not been studied thus far.

Herein, we have characterized the impact of associative learning on the spike timing-dependent plasticity in the vertical pathway between layer IV and layer II/III of mouse barrel cortex and used MMP inhibitors to test whether t-LTP and t-LTD at layer IV-II/III synapses are dependent on activity of these enzymes. We found that learning in classical conditioning paradigm practically prevents t-LTP but has no effect on t-LTD. In addition, applied here, classical conditioning resulted in a strong downregulation of GABAergic tonic currents in the layer II/III interneurons but not in pyramidal neurons. Moreover, blockade of MMP-9 (but not MMP-3) abolishes t-LTP having no effect on t-LTD. We conclude that STDP is strongly correlated with associative learning and critically depends on the activity of MMP-9*.*

## Materials and Methods

### Animals

Experiments were performed on young adult (3–6 weeks old) male Swiss mice. All animals received food and water ad libitum and were kept in a room with controlled temperature with a natural light/dark (12 h:12 h) cycle. All procedures were approved by the Local Bioethical Committee for Experiments on Laboratory Animals (Decision number 29/2015), and an effort was made to minimize the number of animals used for experiments.

### Experimental Procedures

#### Classical Conditioning Sensory Training

Conditioning procedure was carried out as described previously by Siucinska and Kossut [5]. For 1–2 weeks before training, mice were habituated to immobility in a neck restraint apparatus (10 min/day). Afterwards, animals were divided into three groups. In the first group (conditioned stimulus (CS) + unconditioned stimulus (UCS) group), animals were exposed to CS, which consisted of stroking the row B vibrissae on left side of the snout using a handheld brush. The CS comprised three strokes, each lasting 3 s. During the last second of the third stroke, an electrical shock (UCS, 0.5 mA, 0.5 s) was applied to the tail. After a 6-s gap, the trial was repeated. CS + UCS pairing was repeated four times per minute, 10 min per day for 3 days. The second group of mice was the control group (PSEUDO CS + UCS) in which animals obtained the same pattern of CS as in the CS + UCS group, but UCS was applied at random. In CS + UCS and PSEUDO CS + UCS groups, the number of sessions was the same (10 min/day, for 3 days). The naive group was considered as the second control, and it consisted of mice habituated to a neck restraint apparatus.

#### Preparation of Slices

One day after the end of training procedure, mice were decapitated and their brains were immersed in a cold artificial cerebrospinal fluid (ACSF) bubbled with carbogen (95 % O_2_ + 5 % CO_2_), which contained (in mM) NaCl 119, KCl 2.5, NaH_2_PO_4_ 1, NaHCO_3_ 26.3, Mg SO_4_ 1.3, CaCl_2_ 2.5, and d-glucose 11, pH 7.4 [17]. Brain slices (350-μm-thick) were prepared by cutting orthogonally to the rows of barrels in an oblique coronal plane (55° from the sagittal plane) through the barrel field using a vibrating microtome (Leica VT1200S, Germany) [40]. Then, slices were transferred to a recovery chamber containing the same solution and incubated at room temperature for at least 2 h before electrophysiological experiment or fixation procedures.

#### Electrophysiological Recordings

Individual slices were transferred to a recording chamber where they were minimally submerged and continuously superfused with carbogen-saturated ACSF. A low-power objective (×4) was used to identify barrels within the slice, and a high-power water immersion objective (×40) with infrared differential interference contrast was used to visualize individual neurons located in the layer II/III in the barrel B column (corresponding to the whiskers stroked during training). Whole-cell patch-clamp recordings in current clamp mode were performed from layer II/III pyramidal neurons located above the barrel B using borosilicate patch pipettes filled with the intracellular solution containing (in mM) 116 potassium gluconate, 6 KCl, 2 NaCl, 0.5 EGTA, 20 HEPES, 4 MgATP, 0.3 NaGTP, and 10 Na_2_ phosphocreatine, pH 7.25, 290–300 MOsm [17]. Voltage-clamp recordings were made from layer II/III pyramidal neurons, fast spiking (FS) interneurons, and non-fast spiking (NFS) interneurons at a holding potential −75 mV. Patch pipettes had 3–4 MΩ when filled with the internal solution containing (in mM) 140 KCl, 1 MgCl_2_, 0.5 EGTA, 10 HEPES, and 4 MgATP, pH 7.3, 290–300 MOsm [13]. All recorded signals were low-pass-filtered at 10 kHz using the eight-pole Bessel filter built within Multiclamp 700B patch-clamp amplifier (Molecular Instruments, Sunnyvale, CA, USA), digitized at 20 kHz (Digidata 1440, Molecular Devices), and acquired with pClamp 9.2 software (Axon Instruments, USA). Series resistance was monitored, and when it changed by more than 20 % during recordings, cells were rejected.

Classification of neuron type was based mainly on firing pattern described previously by Avermann et al. [41]. Pyramidal neurons in layer II/III were first visually distinguished by a prominent apical dendrite and a pyramidal shape of somata. These neurons were further identified as excitatory, based on their broad action potentials (APs half-width >1 ms), relatively high input resistance (*R*_ind_ ∼ 140 MΩ, *R*_ind_—input resistance of neuron obtained from responses to small depolarizing current steps, +25 pA), and adapting firing patterns. Interneurons in layer II/III were divided into two populations: fast spiking and non-fast spiking inhibitory neurons. Several electrophysiological features differentiated these two classes of layer II/III GABAergic neurons. First of all, FS neurons generated firing patterns with narrow AP waveforms and little adaptation while NFS interneurons had broad APs. Secondly, in agreement with previous observations by Avermann et al. [41], the membrane time constant in response to hyperpolarizing current injection was faster for FS (*τ* ∼ 8 ms) than for NFS interneurons (*τ* ∼ 18 ms). An important clue to distinguish excitatory cells, FS interneurons, and NFS neurons from each other came from the analysis of the somatic input resistance. NFS interneurons showed high (*R*_ind_ ∼ 260 MΩ) whereas FS lower *R*_in_ values (*R*_ind_ ∼ 130 MΩ). Intrinsic electrophysiological properties of neurons were evaluated from the response to 300 ms current injection incrementing by 25 pA, starting at −200 pA. The input resistance of neuron was calculated from responses to small hyperpolarizing current steps (−25 pA, *R*_inh_) and depolarizing ones (+25 pA, *R*_ind_). The resting membrane potential (*V*_m_) of neuron was measured within 15 ms time window at which no current stimuli were applied. The membrane time constant (*τ*) was calculated from a single exponential fit to the rising phase of responses to small hyperpolarizing (−25 pA) current stimulus. Action potential threshold (AP threshold), action potential amplitude (AP amplitude), action potential half-width (AP half-width), and fast afterhyperpolarization (fAHP) were measured at the smallest current stimulus that evoked spikes. AP threshold was defined as a membrane potential value at which dV/dt = 20 mV/ms. AP amplitude was determined as a difference between AP threshold and the peak of the AP. The AP half-width was calculated as the width of the AP at half-maximal amplitude. fAHP was defined as a difference between the threshold and the most negative membrane potential immediately succeeding the spike. Several steps were taken to determine the gain and firing threshold. First of all, the mean firing rate was estimated as the number of action potentials evoked by current injection per 300 ms (pulse length). This parameter was calculated for a series of current steps of increasing amplitude, starting from −200 pA with 25 pA increments. Next, we plotted the firing rate–current relationship for recorded traces. Ultimately, the mean gain was determined as the slope of the linear fit to the firing rate–current curve and firing threshold was defined as a current extrapolated at zero firing rate when first spike was induced.

To induce spike timing-dependent plasticity, a bipolar concentric stimulation electrode (125 μm, FHC, USA) was placed within the base of barrel B in layer IV and excitatory postsynaptic potentials (EPSPs) were evoked at constant rate of 0.1 Hz. Such baseline signals (located in layer II/III within the same barrel column) were recorded from the postsynaptic neurons in the current clamp mode for at least 7 min with the stimulus intensity adjusted to evoke small, single-component EPSPs. After stable baseline recordings, the EPSP was paired 100 times (0.1 Hz) with a single postsynaptic action potential, which was evoked by somatic current injection. To induce t-LTP, the postsynaptic action potential was evoked 10 ms after the onset of the EPSP (forward pairing protocol), whereas to induce t-LTD, the postsynaptic action potential was evoked 10–15 ms before the onset of the EPSP (reverse pairing protocol) [17]. Presynaptic stimulation frequency remained constant throughout the experiment. The slope of the EPSP was calculated as a linear fit between time points on the rising phase of the EPSP corresponding to 20 and 80 % of the positive peak response. Both EPSP peak amplitudes and slopes were monitored for at least 35 min after each pairing episode. The average EPSP slope or amplitude during the baseline was estimated from 30 consecutive sweeps immediately before the plasticity induction, and the same calculation was performed from 30 sweeps postpairing (starting 30 min after pairing). The extent of t-LTP or t-LTD was defined as the ratio of the average EPSP slope or amplitude during the baseline and after plasticity induction, and these values were compared among groups (CS + UCS group, PSEUDO CS + UCS group, and naive group). Investigation of STDP at the vertical layer IV input onto layer II/III was restricted to pyramidal neurons. This protocol has not been applied for interneurons in this pathway for two main reasons. First, Lu et al. [42] showed that when measuring from FS interneurons in the layer II/III of the somatosensory cortex, only t-LTD (at PC-FS synapses) could be induced. Second, besides FS class, we have considered the NFS interneurons which represent a non-homogeneous group and therefore conclusions based on STDP analysis would be problematic. In the case of experiments performed with MMP inhibitors, the extent of t-LTP or t-LTD was compared between test (brain slices from naive animals treated with suitable MMP inhibitor) and control group (in the absence of drug). The following MMP inhibitors were used: FN-439 (180 μM, Merck), SB-3CT (10 μM), and NNGH (10 μM). The stock solutions of SB-3CT and NNGH were dissolved in dimethyl sulfoxide (DMSO), and therefore, the same amount (0.1 % *v*/*v*) of this solvent was added into the ACSF used for control recordings. All the MMP inhibitors were purchased from Sigma-Aldrich (Poland) except FN-439 (Merck). Since EPSPs showed some variability and observed changes in amplitudes following t-LTP or t-LTD induction were moderate, averaged values of 30 consecutive signals (after pairing) were compared. Thus, comparison was made for signals collected within approximately 5 min interval. The electrophysiology data were analyzed using pClamp 10.2 (Molecular Devices) and Sigma-Plot (Systat Software). The statistical analysis was performed using unpaired *t* test to test the differences between the extent of t-LTP and t-LTD in control and MMP inhibitor-treated group. For analysis of differences in the extent of t-LTP and t-LTD between CS + UCS, PSEUDO CS + UCS, and naive groups, we applied one-way ANOVA test or one-way ANOVA on ranks test. The latter test was used in the case of non-normality. Statistical significance was considered for *t* below 0.05.

Since Bragina et al. [43] have shown that tonic currents in layer II/III pyramidal cells were barely detectable, we used THIP (a superagonist for extrasynaptic δ-subunit-containing GABA_A_Rs) to enhance them see also [44, 45, 13]. In our experiments, the values of GABAergic tonic currents were estimated by the baseline current shift observed after PTX (100 μM) application into the ACSF containing the following drugs: GABA_B_ receptor blocker CGP 55845 {(2S)-3-[[(1S)-1-(3,4-dichlorophenyl)ethyl]amino-2-hydroxypropyl] (phenylmethyl)-phosphinic acid), 1 μM}, blockers of glutamate receptors DNQX (6,7-dinitroquinoxaline-2,3-dione, 20 μM) and APV [(±)-2-amino-5-phosphonopentanoic acid, 100 μM], TTX (block the firing of action potentials, 1 μM), and THIP (20 μm). All the drugs were purchased from Sigma-Aldrich (Poland) except TTX and THIP, which were from Latoxan (Poland) and Tocris Bioscience (UK), respectively. The average tonic currents were normalized to the membrane cell capacitance (*C*_m_) and expressed as absolute current density (pA/pF). The membrane capacitance was calculated as the ratio of the membrane time constant and the input resistance, while membrane time constant was obtained from the exponential fit to the time course of the membrane voltage (in the current clamp mode) in response to injection of a small hyperpolarizing current. For each considered type of neurons (pyramidal neurons, FS interneurons, NFS interneurons), tonic GABAergic currents were recorded in slices from naive, CS + UCS, and PSEUDO CS + UCS groups. Statistical comparisons of electrophysiological data were performed with one-way ANOVA test or one-way ANOVA on ranks test in the case of non-normality. The critical level of significance was set at 0.05.

#### In Situ Zymography and Immunofluorescence

Three hundred fifty-micrometer-thick brain slices were immersed in alcoholic fixative and then embedded in pure wax (Science Services, Munchen, Germany) according to procedure described in detail by Wiera et al. [46]. Four-micrometer-thick sections of fixed, wax-embedded brain tissue were cut on rotary microtome (Leica, RM 2255, Germany) and mounted on Superfrost Plus slides (Menzel Gläser, Germany). For localization of gelatinolytic activity, in situ zymography was performed as described previously by Wiera et al. [46]. First, coronal sections were dewaxed in 99.8 % ethanol (37 °C, 3 × 10 min), rehydrated with distilled water (room temperature, 3 × 10 min), and incubated in tap water (37 °C, 90 min). Next, fluorogenic substrate—DQ-gelatin (1:100 dilution in manufacturer’s buffer, Invitrogen, USA)—was put on the top of each tissue section and incubated in the dark at 37 °C. After 80 min, the sections were rinsed with buffer saline with 0.025 % Triton X-100 (TBS-Tx, three times, 15 min) and then blocked for 1 h with 20 % normal horse serum (NHS, Vector Laboratories, USA), washed with TBS-Tx (3 times, 15 min), and finally incubated overnight (at 4 °C) with 2 % NHS together with diluted primary antibodies against vesicular glutamate transporter 2 (VGLUT2, 1:500, polyclonal, Synaptic System). On the next day, sections were washed with TBS-Tx (three times, 15 min), incubated with diluted secondary antibodies (AlexaFluor 568 donkey anti-rabbit, 1:1000) for 2.5 h at RT, then rinsed using TBS-Tx, and finally mounted with Fluoroshield.

#### Image Acquisition and Analysis

DQ-fluorescence (DQ-FL) was visualized using an Olympus Fluoview 1000S laser scanning confocal microscope (Olympus, Japan) in 4-μm thin sections. The sections were additionally stained for VGLUT2 which is a marker of thalamocortical axon terminals [47]. This staining enabled us to visualize the barrels and to estimate the DQ-fluorescence signals at puncta in the close vicinity of the glutamatergic thalamocortical axon terminals [Fig. 5a, b and 6c]. Then, the analysis was focused on “trained” barrel B (objective × 40), and we acquired the images of the layer II/III at the same cortical column. In the same section, we also imaged the “non-trained” barrels (D and E) and layers II/III located above them using identical acquisition parameters. The mean DQ-FL signal inside the barrels B, D, and E and in the layer II/III of the corresponding cortical columns was analyzed using ImageJ (http://rsb.info.nih.gov/ij/) software. Since fluorescence from cytoplasmic and extracellular compartments were not distinguishable, the mean fluorescence intensity was calculated as an averaged signal following exclusion of nuclei which could be easily distinguished both morphologically and by a high fluorescence intensity (Fig. 5b). The mean DQ-FL signal was also analyzed with respect to the background value, which was determined for each individual section from at least three measurements of fluorescence intensity in areas which contained no clearly compartmentalized structures. To quantify the DQ-FL signals at glutamatergic terminals, sections stained with VGLUT2 were considered. First, images were thresholded using Otsu’s automatic method. Next, the selection of VGLUT2 positive puncta inside the barrels B, D, and E was created and transferred to the DQ-gelatin image (the same image, different wavelength acquisition channel). Finally, mean DQ-FL intensity for the all VGLUT2 positive puncta (i.e., the respective selection) was measured. Statistical analysis was performed using paired *t* test with significance levels of *p < 0.05.

## Results

### Associative Learning Affects Spike Timing-Dependent LTP in the Vertical Pathway Between Layer IV and Layer II/III in the Same Barrel Column

**Fig. 1:**
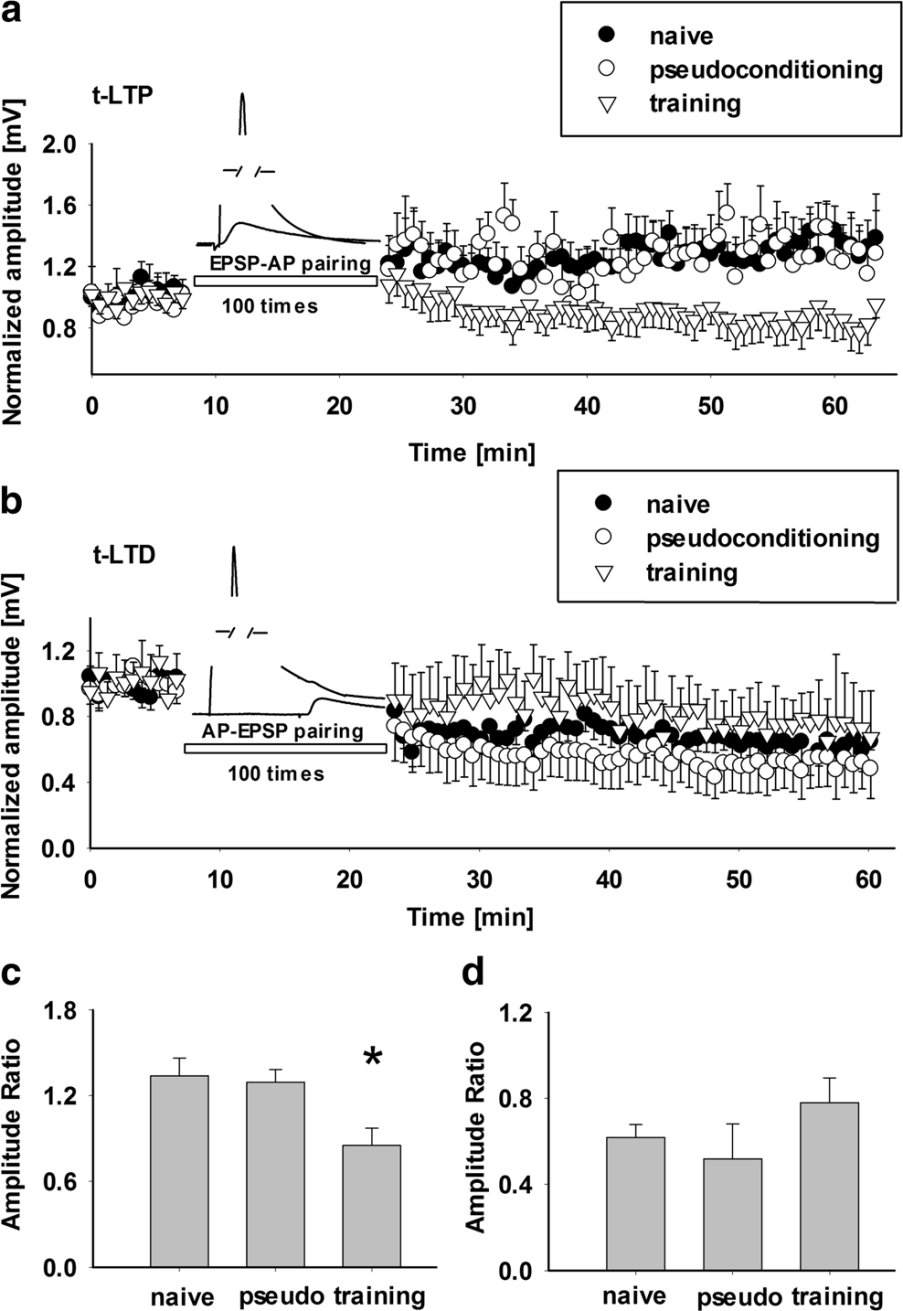
Associative learning prevents t-LTP induced by spike timing-dependent protocol but does not affect t-LTD in vertical pathway from layer IV to layer II/III. **a** Averaged time course of relative EPSP amplitude before and after application of forward pairing protocol inducing t-LTP. Note that there was no difference in the extent of EPSP potentiation between naive (*filled circles*) and pseudoconditioning (*open circles*) groups, but in CS + UCS group of mice (*open triangles*), t-LTP is prevented by classical conditioning with EPSPs showing a tendency to decrease with respect to the baseline values. **b** Averaged time course of relative EPSP amplitude before and after reverse forward pairing protocol inducing t-LTD. Note that behavioral training had no significant effect on the extent of depression in the considered groups (CS + UCS, *open triangles*; naive mice, *filled circles*; PSEUDO CS + UCS group, *open circles*). **c**, **d** Statistics of averaged EPSP amplitude measured at 30–35 min postpairing. *Bars* in **c** represent the mean (± SE) EPSP amplitude ratio in the forward pairing protocol inducing t-LTP (**a**) whereas **d** shows respective statistics for data related to t-LTD induction (**b**) recorded from naive, pseudoconditioned, and trained animals (*Asterisk* indicates significant difference between CS + UCS and both control groups, *p* < 0.05, ANOVA)

First, we have checked whether learning affected the intrinsic electrophysiological properties of pyramidal neurons in layer II/III. We compared the following properties determined for pyramidal cells in slices from CS + UCS versus PSEUDO CS + UCS group as well as cells in slices prepared from naive animals: resting membrane potential *V*_m_, input resistance calculated from responses to small hyperpolarizing current steps *R*_inh_ or depolarizing ones *R*_ind_, AP amplitude, fAHP, AP half-width, mean gain, AP threshold, membrane time constant *τ*, and mean threshold. As shown in Table 1 (Online Resource 1), sensory learning had no significant effect on the properties of excitatory cells in layer II/III (*p* > 0.05, ANOVA on ranks). Thus, we next tested whether the basic properties of the EPSPs evoked by basal stimulation in the vertical pathway between layer IV and layer II/III in the same barrel column showed differences among considered groups (CS + UCS, PSEUDO CS + UCS, naive). The average amplitude and slope of EPSPs during the baseline recordings were 2.34 ± 0.41 mV and 0.8 ± 0.12 mV/ms (*n* = 12), respectively, in PSEUDO CS + UCS group and 2.09 ± 0.21 mV and 0.78 ± 0.05 mV/ms (*n* = 21) in the naive group. Behavioral training did not affect EPSP amplitude or slope (2.26 ± 0.22 mV, 0.82 ± 0.075 mV/ms, *n* = 19, *p* > 0.05, ANOVA on ranks). Thus, associative learning had no effect on considered electrophysiological properties of layer II/III pyramidal neurons or baseline EPSPs recorded in this layer. Since synaptic plasticity is commonly believed to be a substrate for cognitive processes, it can be expected that sensory learning might interfere with long-term plastic changes in the considered model. To verify this possibility, we checked whether behavioral training affected the susceptibility of layers IV to II/III vertical pathway to develop long-term plasticity following application of pairing protocols in the “trained” barrel. To this end, we first checked whether spike timing-dependent LTP was affected by previous classical conditioning. As expected, application of forward pairing protocol induced a stable long-term potentiation in pyramidal neurons in control slices prepared from naive or PSEUDO CS + UCS mice (mean increase in EPSPs was 1.33 ± 0.13, *n* = 9, and 1.29 ± 0.09, *n* = 5, in naive and PSEUDO CS + UCS, respectively, *p* < 0.05, unpaired *t* test, Fig. 1a, c). Intriguingly, in slices from animals which underwent classical conditioning procedure (CS + UCS group), t-LTP was completely abolished showing even a tendency to decreased EPSP values postpairing (0.85 ± 0.12 with respect to baseline level, *n* = 11, Fig. 1a, c). Notably, EPSPs measured in control and CS + UCS groups showed significant difference as early as 15 min after t-LTP induction and remained stable throughout the entire recording period (*p* < 0.05, ANOVA, Fig. 1a). These results clearly indicate that the sensory learning (CS + UCS) has a profound impact on the synaptic plasticity in the considered model by preventing the t-LTP. It is interesting to test whether learning in classical conditioning paradigm affects also t-LTD in the same projection, and to address this issue, t-LTD was induced using the reverse pairing protocol (Fig. 1b, “Materials and Methods”). In all considered groups, t-LTD was successfully induced but there were no significant differences in the average reductions of EPSP amplitude between CS + UCS (0.78 ± 0.12 of baseline levels, *n* = 6), PSEUDO CS + UCS (0.52 ± 0.16 of baseline levels, *n* = 6), and naive (0.62 ± 0.06 of baseline levels, *n* = 8) groups of mice (*p* > 0.05, ANOVA, Fig. 1b, d).

### Sensory Learning Affects Tonic Currents in a Neuron-Specific Manner

**Fig. 2:**
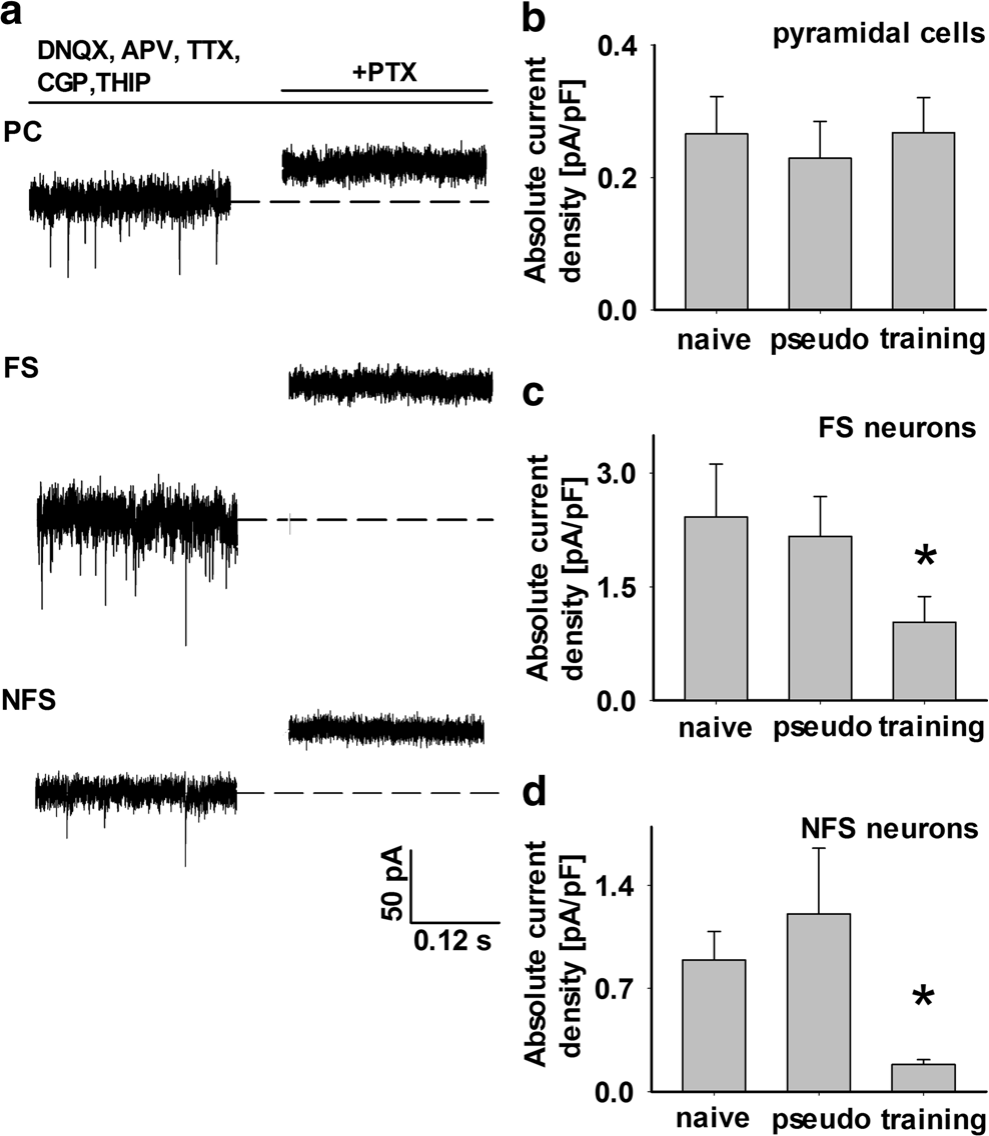
Classical conditioning paradigm affects THIP-elicited tonic currents in layer II/III in a neuron-specific manner. **a** Examples of tonic current recordings from excitatory (*PC* pyramidal cells) and from inhibitory (*FS* fast spiking; *NFS* non-fast spiking) neurons located in layer II/III of slices from naive mouse, evoked by the δ-subunit preferring GABA_A_ agonist THIP. *Bars* above traces indicate applications of drugs specified above. **b**–**d** The mean (± SE) absolute current densities recorded from slices prepared from naïve, pseudoconditioned, and trained animals in layer II/III excitatory cells (**b**) and interneurons (**c** FS neurons, **d** NFS neurons). (*Asterisk* indicates significant difference between CS + UCS and both control groups for the specified type of neuron, *p* < 0.05, ANOVA on ranks)

Since synaptic plasticity at glutamatergic synapses can be affected by the inhibitory drive, it seems interesting to check whether the sensory learning affects inhibition. Interestingly, tonic inhibition was found to be particularly prone to plastic changes upon learning [13], and therefore, we decided to check for the impact of sensory learning on the GABAergic tonic currents in the layer II/III neurons in our model. To this end, tonic currents were elicited by exogenous application of solution containing 20 μm THIP together with agents blocking GABA_B_ receptors, glutamate receptors, and firing of action potentials (see “Materials and Methods”). The amplitude of tonic current elicited by THIP was assessed by blocking it by 100 μM PTX (Fig. 2a). The mean values of tonic currents normalized to membrane capacitance were 0.27 ± 0.06 pA/pF (*n =* 18, pyramidal neurons), 2.41 ± 0.7 pA/pF (*n =* 7, FS), and 0.89 ± 0.19 pA/pF (*n* = 6, NFS) measured from slices prepared from the naive animals. As shown in Fig. 2b, there were no significant differences in the absolute tonic current density measured from pyramidal neurons in any considered experimental group (naive, PSEUDO CS + UCS, and CS + UCS; *p* > 0.05, ANOVA on ranks). In the case of both types of interneurons, there was no difference between tonic current density in the naive and PSEUDO CS + UCS groups (FS neurons = 2.41 ± 0.7 pA/pF, *n* = 7, naive; 2.16 ± 0.53 pA/pF, *n* = 7, PSEUDO CS + UCS; NFS neurons = 0.89 ± 0.19 pA/pF, *n* = 6, naive; 1.21 ± 0.45 pA/pF, *n* = 5, PSEUDO CS + UCS; *p* > 0.05, ANOVA on ranks), but this current in the CS + UCS group showed significantly smaller values than in the two controls (FS neurons = 1.03 ± 0.33 pA/pF, *n* = 4; NFS neurons = 0.185 ± 0.03 pA/pF, *n* = 6; *p* < 0.05, ANOVA on ranks, Fig. 2c, d). Additionally, we have checked whether intrinsic electrophysiological properties of FS interneurons in layer II/III are affected by classical conditioning training. NFS interneurons have not been analyzed because of their heterogeneity. We have not observed any significant changes in properties of FS in our model (data not shown).

Altogether, these results provide evidence that behavioral training strongly downregulates tonic currents in considered here two types of interneurons but has no effect on this current in the pyramidal neurons.

### Spike Timing-Dependent LTP in Layer II/III Barrel Cortex Is Dependent on Metalloproteinase Activity

Previous data reported a crucial role of matrix metalloproteinases in mechanisms of synaptic plasticity, mainly in the hippocampal LTP. It raises a question about the role of these enzymes in regulating the synaptic plasticity in barrel cortex which has not been studied thus far. Thus, the next step was to assess the involvement of the metalloproteinase activity in the induction and consolidation of the spike timing-dependent synaptic plasticity in the considered pathway. To this end, we first checked the impact of FN-439 (Calbiochem, USA), a broad-spectrum inhibitor of matrix metalloproteases, on EPSPs and intrinsic electrophysiological properties of pyramidal neurons in layer II/III and on the basic properties of IV-II/III pathway EPSPs. Under control conditions, the mean baseline EPSP slope and amplitude were 0.82 ± 0.25 mV/ms, *n* = 10, and 1.89 ± 0.23 mV, respectively. FN-439 (180 μM) was applied 15 min before recordings and was maintained throughout the experiment. As shown in Table 2 (Online Resource 1), no changes in properties of pyramidal neurons were observed in the presence of this inhibitor. Moreover, bath application of FN-439 did not influence basal EPSP slope or amplitude (0.82 ± 0.16 mV/ms, *n* = 10, and 2.03 ± 0.28 mV; *p* > 0.05, unpaired *t* test).

**Fig. 3:**
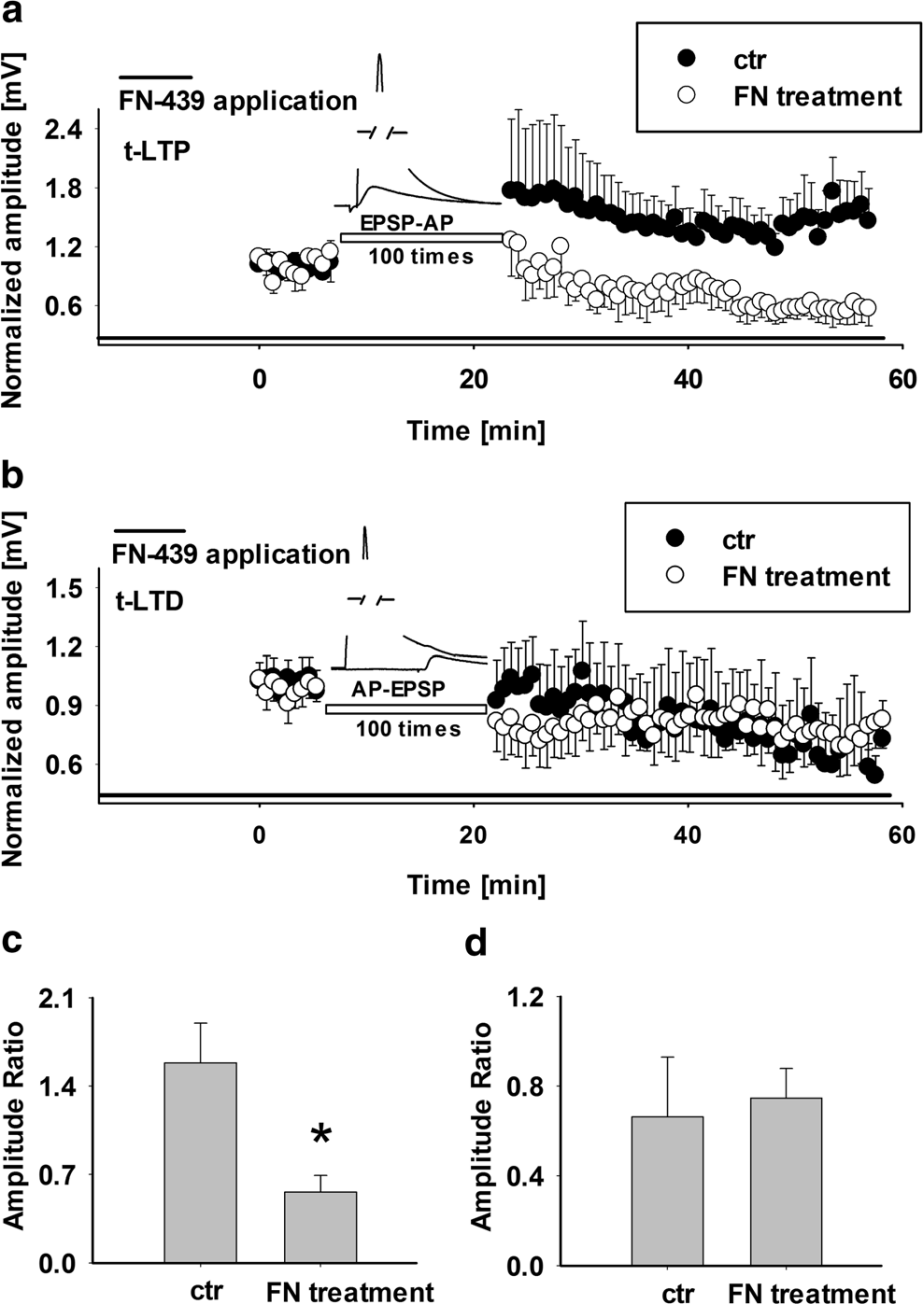
Induction of t-LTP but not of t-LTD in vertical pathway from layer IV to layer II/III of barrel cortex requires MMP activity. **a** Forward pairing protocol induced t-LTP in layer II/III of barrel cortex which is impaired in FN-439 treated slices (*open circles*) in comparison to that in control slices (*filled circles*, **a**). MMP blocker was applied 15 min before the recordings and was present throughout the experiment (*black bar*). **b** Time course of EPSP depression (*t-LTD*) after pairing the EPSP with the somatic current injection in control (*filled circles*) and FN-439-treated slices (*open circles*). Application of FN-439 did not significantly affect the extent of t-LTD. **c**, **d** Statistics for the t-LTP (**b**) and t-LTD (**c**) magnitude (measured at 30–35 min postpairing) recorded from layer II/III pyramidal neurons in control and in FN-439-treated slices. Data are expressed as the mean EPSP amplitude (± SE, *asterisks* indicate significant difference, *p* < 0.05, unpaired *t* test)

**Fig. 4:**
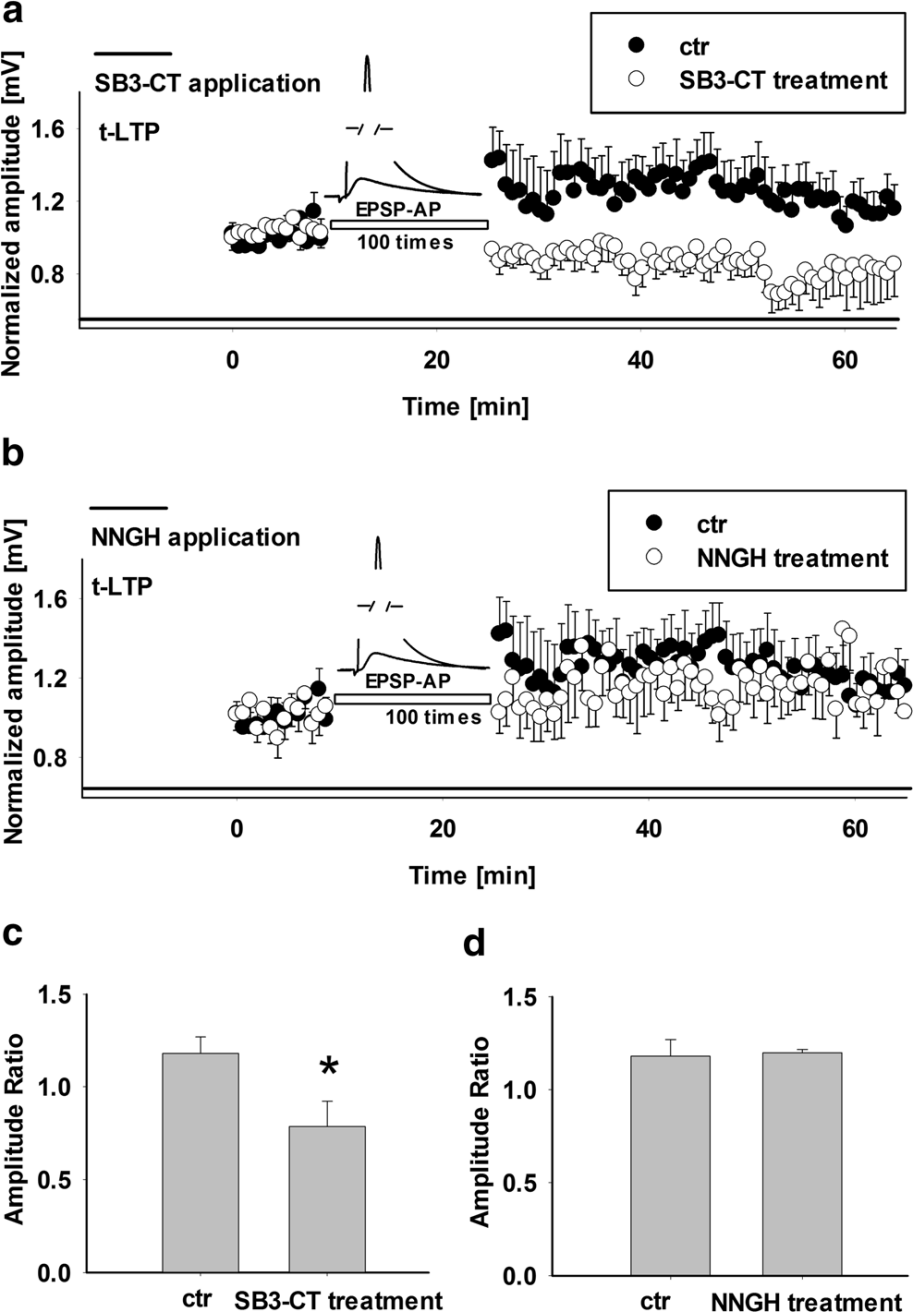
t-LTP is sensitive to SB3-CT (10 μM) but not to NNGH (10 μM) indicating involvement of gelatinases and the lack of effect of MMP-3 in the barrel cortex synaptic plasticity. **a** Time course of relative EPSP amplitude after forward pairing protocol, recorded from control slices (*filled circles*) and from slices treated with MMP-2/9 inhibitor SB3-CT (*open circles*, **a**). Note that t-LTP is suppressed by SB3-CT. MMP-2/9 blocker was added into ACSF 15 min before recordings and was present throughout the recordings (*black bar*). **b** Time course of EPSP potentiation after forward pairing protocol in control slices (*filled circles*) and in the presence of MMP-3 inhibitor NNGH (*open circles*). Application of NNGH caused no significant changes of t-LTP. The *black horizontal bar* represents the application of drug. **c**, **d** Average values of t-LTP measured at 30–35 min after forward pairing protocol. **c** Mean EPSP amplitude ratio recorded from layer II/III pyramidal neurons in control and SB3-CT-treated slices while the average EPSP amplitude ratio in NNGH-treated slices (in comparison to control slices) are presented in **d** (*Asterisk* points out significant difference, *p* < 0.05, unpaired *t* test)

The next step was to check the impact of MMP activity on synaptic plasticity in layer IV-II/III vertical pathway. In control conditions, application of the forward pairing protocol yielded t-LTP with the mean EPSP amplification of 1.58 ± 0.31 (*n* = 7, Fig. 3a, 30–35 min postpairing). Interestingly, superfusion of slices with solution containing FN-439 (starting from 15 min before t-LTP induction) prevented induction of this form of plasticity (Fig. 3a). Moreover, in the presence of FN-439, EPSP amplitude (relative to baseline) showed a progressive reduction and, after approximately 20 min, EPSPs reached 0.62 ± 0.12 of baseline level (*n* = 5, *p* < 0.05, unpaired *t* test, Fig. 3a). As shown in Fig. 3c, the average level of t-LTP at 30–35 min postpairing was reduced to 0.56 ± 0.12 of baseline level (*n* = 5, *p* < 0.05, unpaired *t* test). This observation may suggest that pharmacological blockade of MMPs not only suppress the t-LTP but may shift this system towards LTD. Taking this into account, we checked whether t-LTD was sensitive to blockade of these enzymes and reverse timing-dependent plasticity protocol was used to induce t-LTD in the layer II/III in control conditions and in the presence of FN-439. No significant effect of this blocker on the t-LTD characteristics was found (magnitude of t-LTD was 0.66 ± 0.26, *n* = 6, and 0.75 ± 0.13, *n* = 5, in controls and in FN-439 treated slices, respectively, *p* > 0.05, unpaired *t* test, Fig. 3b, d). Thus, in the considered pathway, MMP inhibitor FN-439 impaired t-LTP but had no effect on t-LTD evoked by pairing protocols. An important problem in interpreting our results is that FN-439 is a broad-spectrum MMP inhibitor that at the concentration used in the present study (180 μM) inhibits MMP-1, MMP-3, MMP-8, MMP-9, and MMP-2 [48] raising thus a question which MMPs are actually involved in regulation of synaptic plasticity in this model. To address this issue, we tested the impact of the following more specific MMP inhibitors: SB-3CT that at concentration used in the present study (10 μM) was found to selectively inhibit MMP-2/9 [49–51] and NNHG that at 10 μM concentration inhibits MMP-3 [52] but also inhibits other MMPs: MMP-1, MMP-8, MMP-12, MMP-13, and MMP-2 [53, 54], according to product data sheet. For the sake of consistency with the previous experiments, we first examined how MMP inhibition affects baseline synaptic transmission and intrinsic electrophysiological properties of LII/III pyramidal neurons. In the case of baseline synaptic transmission, we found no significant effects of the solvent (DMSO at 0.1 % *v*/*v*), SB-3CT, or NNGH (in control conditions, EPSP slope and amplitude were 0.73 ± 0.15 mV/ms and 2.22 ± 0.38 mV, *n* = 7, and the EPSP slopes and amplitudes in the presence of chemical compounds were 1 ± 0.21 mV/ms, 3 ± 0.83 mV (*n* = 6); 0.84 ± 0.23 mV/ms, 1.79 ± 0.23 mV, (*n* = 6); 0.67 ± 0.23 mV/ms, 2.65 ± 0.66 mV, (*n* = 5) for DMSO, NNGH, and SB-3CT, respectively. In addition, intrinsic electrophysiological properties of LII/III pyramidal neurons were not affected by DMSO, NNGH, or SB-3CT treatment (Table 3 and Online Resource 1). Next, we evoked t-LTP by applying forward pairing protocol in slices prepared from control animals in the presence of DMSO (0.1 % *v*/*v*) and found that the extent of t-LTP was stable (1.18 ± 0.09, *n* = 6, Fig. 4a) but apparently smaller than in the case of control determined for ASCF without vehicle (1.39 ± 0.10, *n* = 6). This implies that although DMSO weakens the ability to develop this type of plasticity, stable t-LTP could be still routinely induced. Interestingly, in the presence of selective inhibitor of MMP*-*2 and MMP*-*9, SB-3CT (10 μM), t-LTP was completely abolished (relative EPSP amplitude 0.78 ± 0.14, *n* = 6, *p* < 0.05, unpaired *t* test, Fig. 4a, c). However, application of NNGH did not significantly change the time course or extent of EPSP potentiation and the relative EPSP amplitude potentiation was 1.20 ± 0.018 (*n* = 6, 30–35 min postpairing, *p* > 0.05, in comparison to control, unpaired *t* test, Figs. 4b, d). These results indicate that a critical dependence of t-LTP on MMPs can be attributed to the activity of gelatinases. Notably, MMP-3, known to interfere with synaptic plasticity phenomena in other pathways, appears not to contribute to t-LTP in the present model as NNGH had no effect on the observed plasticity.

### Classical Conditioning Training Is Associated with Increased Activity of Gelatinases in Layer IV of Barrel Cortex

**Fig. 5:**
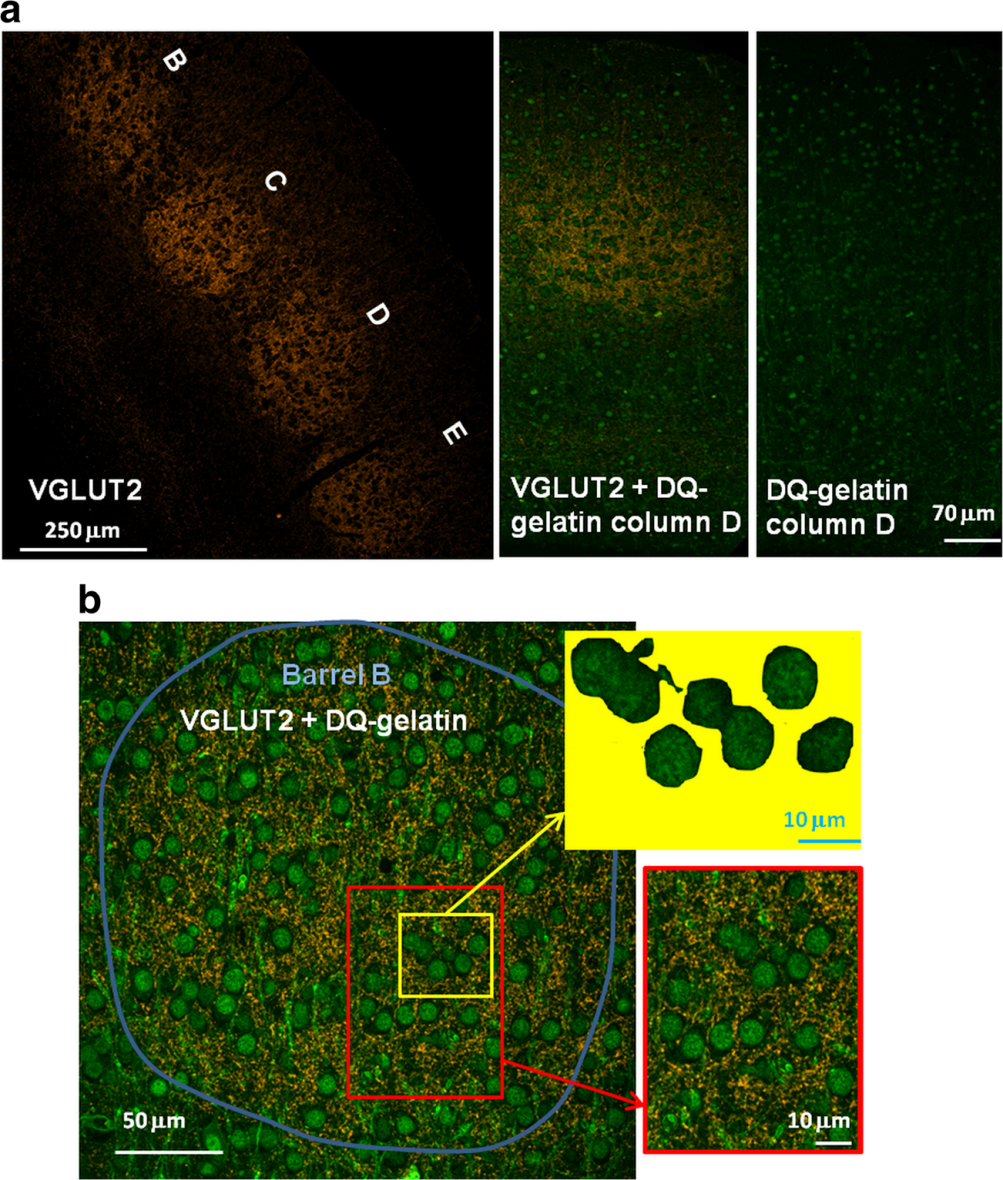
Gelatinolytic activity is assessed by in situ zymography in barrels visualized by immunostaining for VGLUT2. **a** Representative confocal image of a barrel cortex section of mouse (×10 magnification) in brain slices fixed after sensory learning, immunostained for VGLUT2 to visualize the barrels (*left*) and its magnification (*column D*) showing in situ zymography (*green*) and staining against VGLUT2 (*orange*) together (*middle*) and in situ zymography only (*right*). *Letters* mark barrels and the “trained” one is denoted as *B*. **b** Exemplary confocal immunofluorescence image of layer IV barrel cortex (×40 magnification) containing barrel B (indicated with the *blue line*). In situ zymography (*green*) and staining against VGLUT2 (*orange*) are visualized. *Upper inset*: the area (*yellow*) in which DQ-FL intensity was quantified (see “Materials and Methods” for details). *Bottom inset*: a higher magnification image in which VGLUT2-positive puncta (*orange*) and in situ gelatinolysis (*green*) are visible

**Fig. 6:**
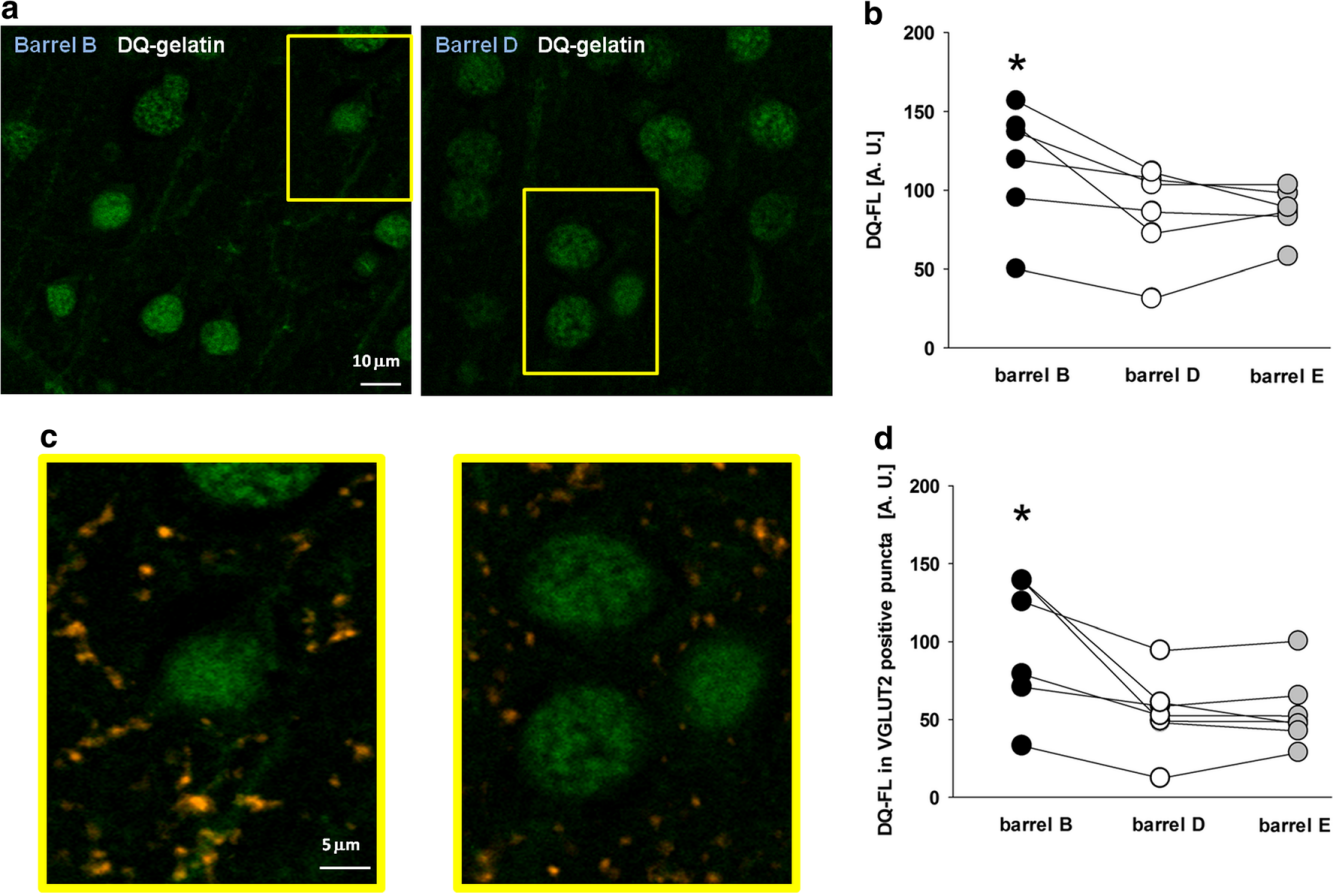
Experience-dependent plasticity in barrel cortex is associated with enhancement of gelatinase activity within the “trained” barrel in layer IV and in the VGLUT2-positive puncta within this barrel. **a** High-magnification images of in situ zymography inside the “trained” barrel B (*left*) and “non-trained” barrel D (*right*). Note a difference in DQ-fluorescence intensities between “trained” and control barrels. *Scale bar*, 10 μm. **b** Statistics of mean DQ-FL intensity of mouse barrel cortex inside the “trained” barrel B (*black circles*) and “non-trained” barrels: D (*white circles*) and E (*gray circles*). Each single *circle* represents the average value obtained for a single slice (*Asterisk* indicates significant difference, *p* < 0.05, paired *t* test). **c** Higher magnification of the images presented in **a** showing in situ zymography (*green*) and staining against VGLUT2 (*orange*) together. *Scale bar*, 5 μm. **d** Statistics of mean DQ-FL intensity in the VGLUT2-positive puncta within the “trained” barrel B (*black circles*) and “non-trained” barrels: D (*white circles*) and E (*gray circles*). Each single *circle* represents the average value obtained for a single slice (*Asterisk* indicates significant difference, *p* < 0.05, paired *t* test)

Since MMPs are known to be strongly involved in formation of memories upon cognitive learning and because, in our model, they are necessary for induction of synaptic plasticity, we assessed gelatinase activity by using in situ zymography on cortical slices collected from animals which underwent the classical conditioning training and from respective controls. For this purpose, the DQ-fluorescence intensity of layers IV and I/III was assessed in 4-μm-thin sections as described in “Materials and Methods and Fig. 5.” We found that behavioral training resulted in a moderate but significant increase in the mean DQ-FL in “trained” barrel B relative to “non-trained” barrel D in layer IV of barrel cortex (1.13 ± 0.04) or relative to “non-trained” barrel E (1.16 ± 0.06; average values in arbitrary units [AU] were 630.4 ± 63.7, 538.9 ± 58.4, and 530.8 ± 71.2 for barrels B, D, and E, respectively; *n* = 6; *p* < 0.05; paired *t* test). Moreover, there were no significant differences in the intensity of fluorescence between “non-trained” barrel E and “non-trained” barrel D (1.03 ± 0.04; *n* = 6; *p* > 0.05; paired *t* test). Considering only the values above the tissue background, the increase in the mean DQ-FL in “trained” barrel B relative to “non-trained” barrel D or E was even larger (B/D = 1.89 ± 0.23, B/E = 1.65 ± 0.29, D/E = 0.93 ± 0.1; average values in AU were 97.8 ± 17.8, 54.6 ± 9.1, and 57.2 ± 8.6 for barrels B, D, and E, respectively; *n* = 6; *p* < 0.05; paired *t* test; Fig. 6a, b). Surprisingly, no changes were observed in the total DQ-FL in layer II/III located above barrels B, E, and D (Online Resource 2).

To see whether there were any changes of the mean DQ-FL in the closest vicinity of glutamatergic terminals, we measured the fluorescence intensity within VGLUT2-positive puncta (Fig. 6c). We observed that, after behavioral training, there was a significant enhancement of the mean DQ-FL in the VGLUT2-positive puncta in “trained” barrel B but not in barrels D and E: B/D = 1.09 ± 0.04, B/E = 1.14 ± 0.06, and D/E = 1.03 ± 0.02 (B/D = 1.36 ± 0.07, B/E = 1.21 ± 0.12, D/E = 0.95 ± 0.09; *n* = 6; *p* < 0.05; paired *t* test). Average values in AU were 649.1 ± 63.5, 555.9 ± 61.7, and 548.1 ± 69.1, for barrels B, D, and E (116.4 ± 15.8, 86 ± 12.4, 87.1 ± 6.5; *n* = 6; *p* < 0.05; paired *t* test; Fig. 6d, values above the background levels). These results indicate that behavioral learning in the considered paradigm is associated with enhancement of gelatinase activity in a manner that is specific for “trained” barrels within VGLUT2-positive puncta in layer IV of barrel cortex.

Importantly, in situ zymography reveals activity of both gelatinases but activity of MMP-2 is localized mainly in nuclei [55], which was excluded from our analysis and therefore the observed fluorescence signal can be ascribed to MMP-9 activity.

## Discussion

The present data demonstrate that LTP induction by STDP protocol shows a strong dependency on prior sensory learning in the classical conditioning paradigm in which vibrissae stimulation is paired with electrical shock to the tail. Strikingly, this behavioral training results in the inability of the system to develop STDP-induced LTP, showing even a trend to reverse the “polarity” of this plasticity to LTD (Fig. 1). It is tempting to explain this observation by proposing that training could occlude the plasticity in the considered pathway. Such an effect was observed, for instance, by Rioult-Pedotti et al. [56] for rat motor cortex where the LTP was reduced and the LTD was enhanced in layer II/III horizontal connections as a result of behavioral training. Moreover, Whitlock and Bear [57] have found that aversive learning was associated with reduced LTP in the CA3-CA1 hippocampal projection, indicating that occlusion of plasticity by behavioral learning could be a widespread feature of neuronal networks occurring also beyond the neocortex. However, observed here changes in plasticity are unlikely to result from a simple LTP occlusion as basal EPSPs in naive (or PSEUDO CS + UCS) and trained (CS + UCS) animals did not show any significant difference. Although the mechanism of this strong interference of behavioral training with STDP-induced LTP is not clear, it is likely to reflect modifications of both excitatory and inhibitory synapses affecting thus complex interplay between excitation and inhibition at the network level and some aspects of these hypothetical scenarios are discussed below.

First of all, STDP protocol applied in this study concerns a complex circuitry of excitatory and inhibitory connections comprising a variety of neurons whose projections show a diversity of plastic properties. It needs to be also stressed that STDP protocol is believed to better mimic naturally occurring activity patterns than a “classic” tetanization and therefore is likely to be more “compatible” to interfere with behaviorally induced plasticity than the latter protocol. Indeed, STDP has been proposed as a possible mechanism for experience-dependent plasticity in the neocortex [22, 58, 59]. Among a variety of possible scenarios whereby behavioral training could affect the susceptibility of the considered system to induce t-LTP, there is a particularly abundant body of evidence for functional and structural changes occurring at synapses in the layer IV. For instance, it has been shown that whisker conditioning led to the appearance of more inhibitory synapses on spines in layer IV of somatosensory cortex and increase in GABA concentration in the presynaptic terminals of these synapses [7, 8]. In general, several lines of evidence indicate that behavioral training strongly upregulates GABAergic system in the barrel cortex, especially in the layer IV, which may affect synaptic plasticity [10, 12, 13, 60]. Investigations of various types of neurons in barrel cortex circuits revealed that associative learning causes multiple plastic changes in a neuron-specific manner. Inside the barrel receiving input from the stimulated whiskers, density of somatostatin-containing interneurons is changing and GABAergic tonic current is upregulated in excitatory cells but it decreases in FS interneurons [11, 13]. Moreover, classical conditioning training affects also phasic GABAergic currents leading to an increase in the mean frequency of sIPSPs in excitatory but not in inhibitory neurons located in “trained” barrel [12]. Thus, available evidence clearly indicates that behavioral training has a major impact on GABAergic inhibition, mainly within the layer IV of the barrel cortex. Due to strong vertical connections within layers, these alterations may provide a potentially potent mechanism controlling induction of plasticity also in the layer II/III. Notably, our morphological observations related to plasticity-induced upregulation of gelatinase activity concern the layer IV while in LII/III it was not found, further supporting a key role of the layer IV in shaping the signaling and thereby the plasticity in the barrel cortex. In the present study, we extend our knowledge on behaviorally induced plasticity by providing the first evidence that classical conditioning results in a strong decrease in GABAergic tonic currents in the layer II/III interneurons but not in pyramidal cells (Fig. 2). It can be suggested that upregulation of inhibitory drive both in layer IV and II/III may represent an efficient control mechanism resulting in tuning the synaptic plasticity in response to whisker conditioning. Decrease in GABAergic tonic currents in the interneurons may have also a strong impact on their excitability and thereby on the activity of principal neurons innervated by these interneurons in this layer. Due to strong and dense connections between FS and excitatory cells, frequency of GABAergic phasic currents in principal neurons will be probably increased as it was observed in layer IV [12]. On the other hand, in the scenario in which a NFS interneuron innervates another GABAergic neuron which, in turn, directly inhibits the pyramidal cell, a decrease in GABAergic tonic current in the NFS neuron (i.e., increase in its excitability) would have a disinhibitory effect on the principal cell. Indeed, for example, bipolar cells located in layer II/III innervate other local interneurons [61]; thus, a decrease in bipolar cell tonic currents would lead to disinhibition of principal neurons. It needs to be recognized that our identification based on the firing pattern is not sufficient to precisely determine the type of interneurons from which recordings were made. However, interneurons classified here as FS GABAergic neurons are likely to be mainly represented by basket cells as they are the largest group among LII/III cortical interneurons [61]. On the other hand, NFS are strongly diversified and we cannot precisely indicate specific types of interneurons involved.

In the light of well-established, abovementioned morphological and functional plasticity in the barrel cortex, involvement of extracellular proteolysis in these processes appears particularly interesting. In the present study, we show for the first time that electrophysiologically induced LTP by STDP protocol in the LII/III excitatory cells critically depends on MMP activity. Thus, in the barrel cortex, dependence of LTP on MMPs shows similarity to previous findings mainly in hippocampus [26, 28, 35, 55, 62, 63] but also in amygdala [29] and in prefrontal cortex areas [27]. It is noteworthy that the impact of MMPs has been demonstrated here for plasticity elicited by STDP which, as already mentioned, is believed to more closely mimic physiological activity patterns than a “classic” tetanization. Intriguingly, LTP dependence on MMPs has been demonstrated here in the model in which the effect of behavioral learning on this plasticity is particularly strong. In the light of this finding, it is not surprising that alteration of MMP activity may potently interfere with some cognitive functions [64] and neuroplasticity phenomena in the barrel cortex. Kaliszewska et al. [38] investigated the model of sensory input deprivation and have shown a marked expansion of the spared row representation in response to exploration of new environment and that this plasticity was associated with an increase in MMP-9 activity, while in MMP-9 KO model, plasticity was found to be reduced. Importantly, Kaliszewska et al. [38] observed that in the MMP-9 knockout mice, a modest but significant decrease in plasticity took place in layer IV and also in LII/III, indicating that in the case of complete suppression of MMP-9 activity, plasticity in LII/III might be also affected. This is consistent with our observation that STDP plasticity in pyramidal cells in the layer II/III was sensitive to MMP-9 blockade. However, activity of MMP-9, estimated by in situ zymography, was not affected by associative learning in this layer (Online Resource 2). Thus, in line with this evidence, observed here sensitivity of t-LTP to specific MMP-9 blocker (SB-3CT) and plasticity-related increase in extra-nuclear gelatinolysis point to the crucial role of MMP-9 in these processes. Impact of MMPs on plasticity in the barrel cortex has been studied also in a pathology model of a photothrombotic stroke which has been demonstrated to disrupt use-dependent plasticity in the cortex neighboring to the infarct site [65] but inhibition of MMPs counteracted this impairment [37]. MMPs have been also shown to be involved in the neuroplasticity in other cortical regions. Spolidoro et al. [66] have revealed that inhibition of MMPs prevents the potentiation of non-deprived eye responses to monocular deprivation. More recently, Aerts et al. [36] provided evidence that genetic inactivation of MMP-3 affected the structural integrity and plasticity of the mice visual cortex. Thus, our findings appear consistent with growing evidence that MMPs play a pivotal role in shaping both structural and functional plasticity and related cognitive functions of the cortex. However, in the present study, pharmacological evidence indicates that observed effects are primarily due to involvement of MMP-9, whereas MMP-3 blockade is ineffective, suggesting that involvement of particular MMPs is pathway specific. In the hippocampus, both electrophysiologically induced plasticity as well as memory and learning have been shown to depend on MMP-9 and MMP-3 [26, 30, 35, 62, 67, 68] but precise contributions of these enzymes to specific hippocampal pathways remain to be determined. An important finding of the present study is that behavioral training, besides affecting the ability of considered pathway to develop plasticity, upregulated the activity and expression of MMP-9, although this effect was restricted to the layer IV. As already mentioned above, this observation could suggest that observed here impact of behavioral training on LTP originates from complex network alterations with a strong (if not predominant) contribution from the layer IV. Moreover, following training, we observed a significant enhancement of gelatinolytic activity in VGLUT2-positive puncta which further indicates that plasticity of these synapses is strongly related to the perisynaptic activity of gelatinases.

However, whether or not MMPs are involved in GABAergic structural and functional plasticity, which was found to be particularly prominent in the cortical plasticity, still awaits a systematic study. In general, in-depth investigation of GABAergic and glutamatergic plasticity is needed to precisely describe plasticity-induced reorganization of cortical circuits [69–71]. The mechanisms underlying structural and functional rearrangements of neuronal circuits are far from being understood, but in the past decade, extensive body of evidence accumulated that processing of extracellular matrix by MMPs is involved in these processes [63, 72–74].

In conclusion, our data show that t-LTP in barrel cortex is abolished in trained mice and that inhibition of MMP-9 activity disrupts STDP in this area. Additionally, considered here behavioral learning is associated with plasticity of the GABAergic tonic inhibition. We propose that these observations reflect MMP-dependent reorganization of cortical circuits.

## Electronic supplementary material

ESM 1 (PDF 166 kb)

ESM 2 (PDF 291 kb)
