# Supplementary material for: Spike Timing-Dependent Plasticity in the Mouse Barrel Cortex Is Strongly Modulated by Sensory Learning and Depends on Activity of Matrix Metalloproteinase 9
Source: Mol Neurobiol. 2016 Oct 15;54(9):6723–36. doi: 10.1007/s12035-016-0174-y (PMC5622912; doi:10.1007/s12035-016-0174-y)
Supplement: Supplementary file 1 — (PDF 166 kb) [file 12035_2016_174_MOESM1_ESM.pdf]

Spike timing-dependent plasticity in the mouse barrel cortex is strongly modulated by sensory learning and depends on activity of matrix metalloproteinase 9.

Molecular Neurobiology

Katarzyna Lebida[1]*, Jerzy W. Mozrzymas[1,2]

[1]Laboratory of Neuroscience, Dept. Biophysics, Wroclaw Medical University, Wroclaw, Poland

[2]Department of Animal Molecular Physiology, Institute of Experimental Biology, Wroclaw University, Wroclaw, Poland

*** Corresponding author:** Katarzyna Lebida, Email: katarzyna.lebida@umed.wroc.pl

Table 1 Intrinsic electrophysiological properties of excitatory neurons recorded in LII/III of naive, PSEUDO CS + UCS and CS + UCS animals

	Naive (n = 19)	Pseudo CS + UCS (n = 14)	CS + UCS (n = 18)
V_m (mV)	-75.29 ± 1.75	-75.68 ± 1.71	-75.41 ± 1.54
R_{inh} (MΩ)	121.17 ± 5.9	119.44 ± 6.9	118 ± 7.24
R_{ind} (MΩ)	136.69 ± 6.8	131.93± 6.67	134.6 ± 7.49
AP amplitude (mV)	77.73 ± 1.67	75.17 ± 1.62	78.27 ± 1.66
fAHP (mV)	-10.39 ± 0.76	-9.88 ± 0.6	-9.93 ± 0.8
AP half-width (ms)	1.26 ± 0.08	1.34 ± 0.04	1.4 ± 0.1
Mean gain (Hz/pA)	0.15 ± 0.01	0.16 ± 0.01	0.14 ± 0.01
AP threshold (mV)	-35.78 ± 1.37	-33.51 ± 0.97	-35.21 ± 1
τ (ms)	14.83 ± 1.11	16.14 ± 1.58	16.27 ± 1.15
mean threshold (mV)	165.71 ± 11.86	179.86 ± 13.76	170.58 ± 14.59

Table 2 Intrinsic electrophysiological properties of LII/III pyramidal neurons recorded from control slices and slices treated by matrix metalloproteinases inhibitor FN-439

	control (n = 13)	FN-439 treatment (n = 14)
V_m (mV)	-74.25 ± 0.65	-74.18 ± 1.37
R_{inh} (MΩ)	121.94 ± 6.75	125.58 ± 11.7
R_{ind} (MΩ)	140.21 ± 9.78	146.36 ± 12.43
AP amplitude (mV)	73.2 ± 1.7	71.12 ± 1.54
fAHP (mV)	-10.78 ± 1.01	-12.04 ± 1.06
AP half-width (ms)	1.1 ± 0.06	1.17 ± 0.06
Mean gain (Hz/pA)	0.21 ± 0.01	0.2 ± 0.015
AP threshold (mV)	-35.38 ± 1.04	-33.33± 1.18
τ (ms)	15.54 ± 1.02	15.38 ± 1.16
mean threshold (mV)	190.76 ± 15.73	221.81 ± 22.09

Table 3 Intrinsic electrophysiological properties of LII/III pyramidal neurons recorded from control slices and slices treated by matrix metalloproteases inhibitors (SB-3CT, NNGH)

	control (n = 8)	control DMSO (n = 8)	SB-3CT treatment (n = 8)	*NNGH* treatment (n = 7)
V_m (mV)	-72.17± 1.57	-71.19 ± 3.53	-70.84 ± 1.71	-75.3± 1.55
R_{inh} (MΩ)	115.81 ± 11.8	119.67 ± 13.32	117.35 ± 12.78	128.18 ± 15.24
R_{ind} (MΩ)	139.64 ± 18.85	138.91 ± 17.37	129.39 ± 14.49	148.25 ± 21.99
AP amplitude (mV)	76.72 ± 3.48	75.52 ± 3.24	74.06 ± 5.15	76.09± 4.62
fAHP (mV)	-8.84 ± 0.69	-9.6 ± 1.24	-7.67 ± 0.84	-7.96 ± 0.86
AP half-width (ms)	1.44 ± 0.14	1.27 ± 0.5	1.38 ± 0.09	1.34 ± 0.16
Mean gain (Hz/pA)	0.15 ± 0.01	0.17 ± 0.02	0.15 ± 0.02	0.15 ± 0.01
AP threshold (mV)	-32.12± 1.47	-33.61± 1.44	-30.96 ± 2.19	-34.81 ± 1.86
τ (ms)	12.43 ± 1.4	14.69 ± 1.77	12.96 ± 1.66	13.85 ± 0.79
mean threshold (mV)	190.31 ± 25.08	177.96 ± 32	184.93 ± 20.29	152.13 ± 25.04
