# Supplementary material for: Spike Timing-Dependent Plasticity in the Mouse Barrel Cortex Is Strongly Modulated by Sensory Learning and Depends on Activity of Matrix Metalloproteinase 9
Source: Mol Neurobiol. 2016 Oct 15;54(9):6723–36. doi: 10.1007/s12035-016-0174-y (PMC5622912; doi:10.1007/s12035-016-0174-y)
Supplement: Supplementary file 2 — (PDF 291 kb) [file 12035_2016_174_MOESM2_ESM.pdf]

Spike timing-dependent plasticity in the mouse barrel cortex is strongly modulated by sensory learning and depends on activity of matrix metalloproteinase 9.

Molecular Neurobiology

Katarzyna Lebida[1]*, Jerzy W. Mozrzymas[1,2]

[1]Laboratory of Neuroscience, Dept. Biophysics, Wroclaw Medical University, Wroclaw, Poland

[2]Department of Animal Molecular Physiology, Institute of Experimental Biology, Wroclaw University, Wroclaw, Poland

*** Corresponding author:** Katarzyna Lebida, M.Sc., Laboratory of Neuroscience, Department of Biophysics, Wroclaw Medical University, Chalubinskiego 3a, Wroclaw, 50-368, Poland. Email: katarzyna.lebida@umed.wroc.pl,

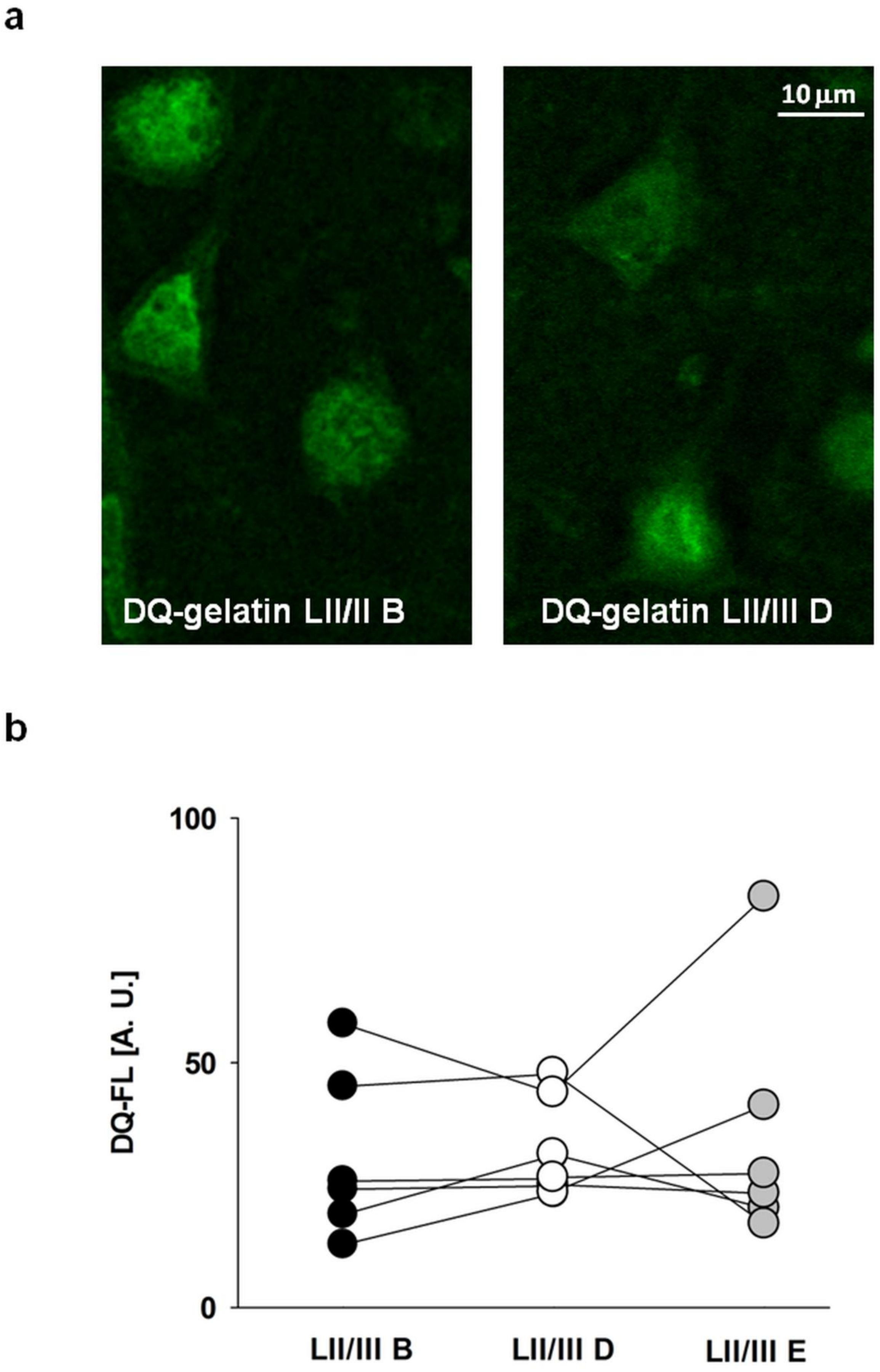
a
10 µm
DQ-gelatin LII/II B
DQ-gelatin LII/III D
b
100
50
0
DQ-FL [A. U.]
LII/III B
LII/III D
LII/III E

Online Resources 2 Associative learning does not affect gelatinases activity in the layer II/III of mouse barrel cortex.

a High magnification images of *in situ* zymography in the layers II/III located above the "trained" barrel B (left) and above "non-trained" barrel D (right). Scale bar – 10 µm.

b Statistics of mean DQ-FL intensity of mouse barrel cortex in the layers II/III situated above the "trained" barrel B (black circles) and "non-trained" barrels: D (white circles) and E (grey circles). Each single circle represents the average value obtained for a single slice.
